# The Role of Moisturizer Containing Anti-inflammatory on Skin Hydration in Mild-Moderate Atopic Dermatitis Patients

**DOI:** 10.1155/drp/3586393

**Published:** 2024-12-23

**Authors:** Cita Rosita Sigit Prakoeswa, Damayanti, Sylvia Anggraeni, Menul Ayu Umborowati, Maylita Sari, Made Putri Hendaria, Tanziela Firdausi Thahir

**Affiliations:** ^1^Department of Dermatology, Venereology and Aesthetic, Dr. Soetomo General Academic Hospital, Surabaya, Indonesia; ^2^Department of Dermatology, Venereology and Aesthetic, Faculty of Medicine, Universitas Airlangga, Surabaya, Indonesia

**Keywords:** anti-inflammatory, atopic dermatitis, eczema, human and health, moisturizers, skin hydration

## Abstract

Atopic dermatitis (AD) is a chronic, inflammatory skin condition characterized by eczema lesions and dry, itchy skin. Recent guidelines for the management of AD emphasize the importance of using moisturizers in the management of AD. This study is a double-blind clinical trial to determine the effectiveness of moisturizers containing anti-inflammatory ingredients compared with moisturizers without anti-inflammatory ingredients for skin hydration in mild to moderate adult AD patients for 14 days at the Dermatology and Venereology Outpatient Clinic at Dr. Soetomo General Academic Hospital. There was a significant difference (*p* < 0.05) at the baseline and day 14 skin hydration values in the experiment group with anti-inflammatory ingredients (35.97 ± 6.04–66.06 ± 15.84) and the control group without anti-inflammatory ingredients (40.74 ± 10.94–56.12 ± 8.34). After comparison, there was a significant difference (*p* < 0.05) in the skin hydration value between the experiment group and the control group on the 14th day. There was also a significant difference in the improvement of skin hydration outcomes between both groups (*p* < 0.05). The severity of the disease using Scoring Atopic Dermatitis (SCORAD) showed a significant difference (*p* < 0.05) between the experiment group and the control group after 2 weeks of intervention. The addition of anti-inflammatory ingredients in the moisturizer, namely, shea butter, bacterial lysate, allantoin, bisabolol, *Phragmites kharka* extract, *Poria cocos*, and *Mirabilis jalapa* in a moisturizer containing occlusive (dimethicone), humectants (glycerin, saccharide, butylene glycol, and hyaluronic acid), and emollient (shea butter and squalane) was shown to be significantly better in improving skin hydration in patients with mild to moderate AD.

## 1. Introduction

Atopic dermatitis (AD) is a chronic inflammatory skin condition characterized by eczema lesions and dry skin accompanied by itching. Globally, the prevalence of AD is quite high as many as 17.1% of adults and 22.6% of children were diagnosed with AD, with 9.6% of the new cases in children every year [1]. The management of AD aims to prevent recurrence, improve the skin barrier, maintain hydration of the stratum corneum, and reduce inflammation [2, 3]. Due to skin barrier dysfunction in AD patients, skin hydration is an important parameter in the evaluation of AD patients [3]. Local steroids used to treat AD have both local and systemic side effects [4]. One of the important components in the management of AD is the use of moisturizers [3]. Moisturizer aids in the repair of epidermal damage and the prevention of AD flare-ups [2]. Studies that discuss the use of moisturizers containing effective anti-inflammatory agents in patients with AD are still limited. The purpose of this study is to compare the clinical improvement in skin hydration of mild to moderate AD patients using moisturizers containing occlusive (dimethicone), humectant (glycerin, saccharide, butylene glycol, and hyaluronic acid), emollients (shea butter and squalane), and anti-inflammatory ingredients (shea butter, bacterial lysate, allantoin, bisabolol, *Phragmites kharka* extract, *Poria cocos*, and *Mirabilis jalapa*).

## 2. Materials and Methods

**Figure 1:**
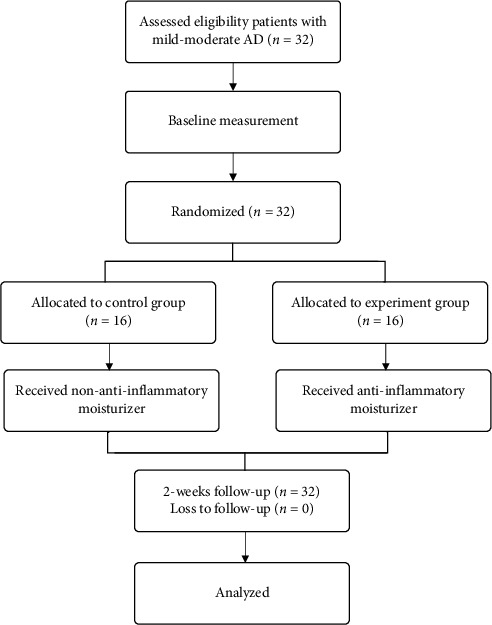
Study flow diagram for the randomized trial.

This study was a double-blind, randomized trial comparing the experimental group and the control group. This research involved 32 patients with mild to moderate AD, aged 18–64 years, who came to the Dermatology and Venereology Outpatient Clinic at the Dr. Soetomo General Academic Hospital. The inclusion criteria for this study were mild-moderate adult AD patients aged 18–64 who met the Hanifin-Rajka criteria for AD diagnosis, whose general condition was good, and who were willing to participate in the study and sign the informed consent. Subjects who met the criteria were randomly divided into the experimental group and the control group, as seen in Figure 1.

The experiment groups include 16 people given moisturizer containing occlusive (dimethicone), humectants (glycerin, saccharide, butylene glycol, and hyaluronic acid), emollients (shea butter and squalane), and anti-inflammatory ingredients (shea butter, bacterial lysate, allantoin, bisabolol, *Phragmites kharka* extract, *Poria cocos*, and *Mirabilis jalapa*). The control groups, which also included 16 people, received moisturizer that contained occlusive (dimethicone), humectants (glycerin, saccharide, butylene glycol, and hyaluronic acid), and emollients (shea butter and squalane) without anti-inflammatory ingredients. Paragon Technology and Innovation carried out the formulation and labeling processes for the control and experimental moisturizers. Both the subject and the researcher did not know the contents of the experimental moisturizer received by each group. Each subject was given the moisturizer and instructed to apply it twice a day to their arms and legs. Evaluation of skin hydration was performed using a Courage Khazaka Cutometer MP-580 that consists of a corneometer to assess stratum corneum hydration levels. We also evaluate the severity of the disease using Scoring Atopic Dermatitis (SCORAD) between the experimental group and the control group. The evaluation was performed three times during the study. The first evaluation was before the experiment as the baseline value, the second evaluation was after 1 week of the experiment, and the last evaluation was at the end of 2 weeks of the experiment. The Ethical Committee of Dr. Soetomo General Academic Hospital Surabaya assessed and approved this study (no. 0356/KEPK/I/2022).

## 3. Results

This study included 32 participants with mild to moderate AD who came to the Dermatology and Venereology Outpatient Clinic at Dr. Soetomo General Academic Hospital and met the inclusion criteria of this study. Subjects were female and male, aged 18–64 years, and suffered from skin dryness but had no clinical manifestation of contact dermatitis. Detailed demographic data are shown in Table 1. On the subject's first visit, the means of skin hydration in the experiment group (inner arm) were 35.97 ± 6.04, and the means of skin hydration in the control group (inner arm) were 40.74 ± 10.94. There is no significant difference (*p*=0.165; CI 95%) in baseline skin hydration between the experimental and control groups.

Based on the analysis conducted, it is known that there is a significant difference (*p* < 0.05) between baseline corneometry values and day 14 for each experiment and control group. After comparison, there was a significant difference (*p* < 0.05) in the skin hydration value between the experiment and control group on the 14th day (Tables 2 and 3).

This study showed that there was a significant difference (*p* < 0.05) in the reduction of the clinical severity between the experiment group and the control group in the second week. There is also a significant improvement in skin hydration shown by the changes in corneometry measurements presented in Table 4. In the experiment group, the SCORAD decreased by 12.90% in the second week compared with the first week and 24.50% in the second week compared with the baseline (Table 5).

## 4. Discussion

The most prevalent form of inflammatory skin disease, AD, affects up to 20% of children and may persist into adults. Barrier disruption is recognized as a significant part of the pathophysiology of AD and correlated with the condition's intensity. The most prevalent symptom and distinguishing feature of AD is pruritus, which is defined as an unpleasant feeling that causes a desire to scratch. Patients with AD suffer from pruritus, which has a detrimental effect on their quality of life by frequently causing sleep disruption, concentration issues, and social withdrawal. It is crucial to keep in mind that pruritus causes an itch-scratch cycle that compromises the epidermal barrier and amplifies the inflammatory response, which feeds the illness [5, 6].

As a result of the intricate interactions between genetic, environmental, and immunologic variables that lead to AD, the illness can manifest in a variety of clinical phenotypes that are categorized based on a wide range of factors, including age of onset, morphology, topography, severity, and disease course. Many clinical variants of AD have been described based on the age of the patient: infantile AD (3 months/2 years), childhood AD (2–12 years), adolescent/adult AD (12–60 years), and elderly AD (> 60 years) [7]. Compared with children and young adults, older individuals have a lower prevalence of AD (1.86%; age ≥ 60) compared with 5.3% (age 6–12) and 3.02% (age 20–44) [8]. Morphology-related clinical phenotypes can be differentiated into nummular, prurigo nodularis, erythrodermic, lichenified, and follicular/popular phenotype [7]. AD is an eczematous disorder, which can also be classified as acute and chronic. Acute and chronic eczema may easily overlap because of the recurrent nature of the disease. Scratching causes excoriations which results in superficial scars. Acute worsening of erythema and itch with abundant oozing may be a sign of superinfection. Chronic lichenified eczema associated with dry skin is the most common presentation [9]. Some topography-related clinical phenotypes examples are head and neck, nipples, hand, and foot. One type of AD affecting the body's seborrheic areas (the head, face, neck, and upper trunk) is head and neck dermatitis. It has a noticeable preference for the eyelids and lips. There is ongoing discussion over the etiology of head and neck AD, with the involvement of Malassezia spp. being one suggested factor [10]. In this study, we conducted an experiment on the subjects' arms.

A number of different therapeutic approaches for AD have been developed, and these include the promotion of skin hydration, the use of emollients, the avoidance of allergens, and the use of antihistamines or corticosteroids during the exacerbation period of the disease [11]. Topical calcineurin inhibitors, such as tacrolimus and pimecrolimus, are anti-inflammatory treatments for AD, both for short and long-term treatments in adults and children older than 2 years of age. TCI can be the first line of therapy for AD in critical locations such as the face, folds, and genitals. TCIs are recommended in maintenance therapy even after corticosteroids have been prescribed [12]. According to the five pillars of AD management outlined by the Asia Pacific consensus, medical therapy should focus on preserving the skin barrier, reducing inflammation, and managing pruritus. Because moisturizing hydrates skin and restores epidermal barrier function, it is regarded as the first stage in treating AD. In addition, moisturizers might lessen itching, lower the need for corticosteroids, and stop flare-ups [5]. According to a study, moisturizers can lessen the severity of SCORAD in AD [13].

Basic emollient therapy is the essence of every treatment of AD/AE. Emollients usually contain humectant or moisturizer to promote stratum corneum hydration, such as urea or glycerol and an occludent to reduce evaporation, such as lipids or petrolatum. A Cochrane review found that emollients containing moisturizers are better at reducing severity and led to fewer flares, as well as a reduction in corticosteroid use [14]. In another study, evidence was found to support the superiority of glycerol-based moisturizers over moisturizers without humectants in order to restore skin hydration [15]. We utilized moisturizers in this study that contain occlusive, humectant, and emollient, which results in a more potent and the moisturizing effect to that of a single mechanism. This is demonstrated by the increase in skin hydration following treatment. In order to effectively control AD, improving skin moisture is crucial. Regular application of a moisturizer will increase skin hydration, lessen the severity of the condition, reduce flare-ups, and enhance overall clinical symptoms [5, 13]. In a study trial presenting infants participating in barrier enhancement for eczema prevention study by applying emollient all over the body daily in the first years of life, the result does not show significant effect and benefit in preventing or delaying AD occurrence or its severity [16]. In accordance with our study, using moisturizers with or without anti-inflammatory ingredients led to a clinically significant improvement in skin hydration in adults with mild to moderate AD.

In this study, both groups had improvements in skin hydration. It is suspected because both moisturizers contain occlusive, humectant, and emollient [17]. The two moisturizers given have the same occlusive, humectant, and emollient content and can be classified as anti-inflammatory compounds. For example, humectants in the form of hyaluronic acid and emollients in the form of shea butter and squalene have been shown to have an anti-inflammatory effect [18–21]. Because the stratum corneum layer attracts the water from the deeper skin layer and from the environment, the humectant causes the amount of water contained in the stratum corneum to increase. The humectant prevents the water from escaping from the stratum corneum layer. The hydration content of the skin can be seen to increase as a result of this mechanism [22]. Patients with AD have dry skin because the skin's ability to bind water decreases. Moisturizer is a treatment for AD and is an effective agent for maintaining skin hydration by lowering TEWL. Moisturizers can also attract, hold, and distribute water on the skin layer [23].

It was discovered that the skin's hydration levels in both groups did not differ significantly. This indicates that both groups had similar characteristics and were comparable. The level of skin hydration is lower in the epidermis of AD patients, and this reduction correlates inversely with FLG degradation products (NMF) [24]. The stratum corneum's hydration level is crucial for the skin's proper functionality and appearance. Increased transepidermal water loss in AD leads the stratum corneum to become less hydrated, which then manifests clinically as xerosis and itching. According to research that compared the skin hydration of 50 adult patients with AD to 50 healthy controls, 78% of AD patients were classified as having dry skin. In comparison to healthy controls, individuals with AD had a mean hydration value of 21.6 ± 17.4 as opposed to 29.8 ± 13.5 [25]. Zainal et al. conducted a study in which he assessed the skin hydration of 48 adult patients with AD at 18 anatomical sites, and all sites were reported to have reduced skin hydration levels [26].

Significant differences (*p* < 0.05) were discovered between the baseline hydration value and day 14 of each group experiment and the control group. The experiment group's skin hydration increased from 35.97 ± 6.04 AU to 66.06 ± 15.84 AU, while the control group's increased from 40.74 ± 10.94 AU to 56.12 ± 8.34 AU. This research is in line with research previously conducted in AD patients, which found that the value of local skin hydration in the test area (elbow fold) increased significantly from 20.86 ± 2.53 to 29.10 ± 3.97 on day 7 (*p* < 0.05) in the moisturizer group without anti-inflammatory agents [18]. In addition, Wakeman found that skin hydration significantly increased (*p* < 0.01) in the Suvex group, which contains anti-inflammatory agents, from 36.45 ± 10.51 AU on day 0 to 42.55 ± 12.70 AU on the 14th day [27].

Based on the analysis carried out, we also found significant differences (*p* < 0.05) in the corneometric value between the experiment group and the control group on the 14th day, where the hydration value of the skin after the administration of moisturizer was significantly better in the experiment group compared with the control group; also, in the delta of the corneometric value between the experiment group and the control group, we also found a significant difference in the experiment group. Increased skin hydration values after the administration of moisturizers are higher in the experiment group compared to the control group. Umborowati et al. also reported an increase in skin hydration from 46.91 ± 18.03 AU to 72.23 ± 23.83 AU in the palmar area can be achieved after using anti-inflammatory ingredients contained in moisturizers for 2 weeks (*p* < 0.001; CI 95%) [28].

In this study, we observed a reduction of SCORAD by 13.20% in the first week after the subject was given the anti-inflammatory moisturizers. Then, after completing the 2-week trial, we found a significant difference (*p* < 0.05) in the reduction of SCORAD between the experiment group and the control group. This indicates that moisturizers with anti-inflammatory ingredients can reduce the severity of the disease after 1 week of use and clinical improvement is achieved after 2 weeks of use. The study by Nurasrifah et al. also found a significant decrease (*p* < 0.0001) in SCORAD score in the intervention group, which contained anti-inflammatory agents, from 20.39 ± 3.69 AU on day 0 to 10.11 ± 3.51 AU on day 14 [13].

The moisturizer used in the experiment group contains anti-inflammatory ingredients, whereas the moisturizer used in the control group does not. Both moisturizers provide significant value in increasing skin hydration from the baseline to post-therapy because of their ingredients. Occlusive is an oil substance with the ability to inhibit TEWL by forming a hydrophobic layer on the skin's surface. Humectants are substances with a low molecular weight that have the ability to attract water, while emollients are oil droplet substances that can enter the gaps between desquamated corneocytes in dry skin to trap water so that the skin's softness and flexibility increase. Occlusives, humectants, and emollients contribute to clinical improvement in AD by increasing skin hydration [25].

A study that examined the hydration level of the skin on the back of the hand discovered that anti-inflammatory ingredients such as ceramide and *Centella asiatica* in moisturizers could increase skin hydration [29]. The experiment group was given moisturizer with the addition of anti-inflammatory substances that also play a role in improving skin hydration; among them were shea butter, *Poria cocos*, and allantoin. Shea butter contains tripertene esters, a natural lipid found in the skin, and sebum functions to smooth the skin by filling the gap between peeling corneocytes [2, 30]. Shea butter forms a layer on the skin's surface to physically prevent water evaporation (transepidermal water loss) [17, 31].

In East Asia, *Poria cocos*, an edible basidiomycete that grows on the roots of pine trees, is extensively used as a traditional medicine. The *Poria cocos* extract has been reported to have a variety of biological activities, including antihyperglycemic, anticancer, anti-inflammatory, and immunomodulatory effects [32, 33]. *Poria cocos* contains dehydrotrametenolic acid (DTA), which is a lanostane-type triterpenic acid that has an effect on the skin's defense function in vitro through a regulatory mechanism on human keratinocyte cells (HaCaT cells). DTA increases the microRNA (mRNA) expression of NMF genes, such as HAS-2, HAS-3, and AQP3, in HaCaT cells. DTA also increases the mRNA expression of various keratinocyte differentiation markers, including TGM-1, involucrin, and caspase-14. In addition, protein expressions of HAS-2, HAS-3, and TGM-2 were significantly increased by DTA [34].

Allantoin is a derivative of urea that has keratolytic activity through the mechanism of working on desmosomes. Allantoin helps eliminate the layer of the epidermis that contains excess keratin. Allantoin also serves to stimulate cell division, stimulate epithelization, and accelerate the regeneration process from skin inflammation. In a murine allergic model, the anti-inflammatory activity of allantoin was also detected, and this was demonstrated by a reduction in IgE levels as well as the production of IL-4 and IL-5. It has been demonstrated that moisturizers containing allantoin are effective in reducing the symptoms of mild to intermediate AD [35, 36].

Using moisturizers with or without anti-inflammatory ingredients led to a clinically significant improvement in skin hydration in mild to moderate AD. Adding anti-inflammatory ingredients to moisturizers has been shown to significantly improve skin hydration even further in patients with mild to moderate AD.

## 5. Conclusion

Using moisturizers with or without anti-inflammatory ingredients led to a clinically significant improvement of skin hydration in mild to moderate AD. Adding anti-inflammatory ingredients to moisturizers has been shown to significantly improve skin hydration and improve skin barrier function and also can reduce inflammation in the lesions of patients with mild to moderate AD. Our study was limited to short time interval. Given that AD is a chronic condition, future study should be done on a longer period of time to gain more information about the long-term effects or potential negative effects.

## Figures and Tables

**Table 1:** Demographic and baseline characteristic.

	Mean ± SD, *n* (%)	*p* value
Age (year), mean ± SD	38 ± 11.8	
Gender		
Male	17 (53.1)	
Female	15 (46.9)	
Occupation		
Office worker	15 (46.9)	
College student	13 (40.6)	
Housewife	2 (6.3)	
Retiree	2 (6.3)	
Atopic severity (SCORAD)		
Mild	29 (90.6)	
Moderate	3 (9.4)	
Other medication history		
No	28 (87.5)	
Yes	4 (12.5)	
Personal atopic history		
Allergic rhinitis	20 (62.5)	
Asthma	6 (18.8)	
Allergic rhinitis and asthma	4 (12.5)	
None	2 (6.3)	
Family atopic history		
No	18 (56.4)	
Yes	14 (43.8)	
Skin hydration baseline		
Experiment group	35.97 ± 6.04	0.165⁣^∗∗^
Control group	40.74 ± 10.94	

⁣^∗∗^No significant difference (*p* ≥ 0.05).

**Table 2:** Comparison of skin hydration values (corneometry) before and after treatment.

	Before treatment (mean ± SD)	After treatment (mean ± SD)	*p* value
Experiment group	35.97 ± 6.04	66.06 ± 15.84	0.001⁣^∗^
Control group	40.74 ± 10.94	56.12 ± 8.34	0.001⁣^∗^

⁣^∗^Significant difference (*p* < 0.05).

**Table 3:** Comparison of skin hydration values (corneometry) between groups at day 14.

	After treatment (mean ± SD)	*p* value
Experiment group	66.06 ± 15.84	0.044⁣^∗^
Control group	56.12 ± 8.34	

⁣^∗^Significant difference (*p* < 0.05).

**Table 4:** The improvement in skin hydration values (corneometry) outcome.

	*δ*	*p* value
Experiment group	26.43 ± 3.22	0.006⁣^∗^
Control group	15.37 ± 1.65	

*Note:δ* = delta of skin hydration values (corneometry).

⁣^∗^Significant difference (*p* < 0.05).

**Table 5:** Comparison of reduction in clinical severity scores.

	*δ* baseline–week 1	*δ* week 1–week 2	*δ* baseline–week 2
Experiment group	13.20%	12.90%	24.50%
Control group	12.00%	5.20%	16.70%
*p* value	0.433	< 0.001⁣^∗^	< 0.001⁣^∗^

*Note:δ* = delta of SCORAD.

⁣^∗^Significant difference (*p* < 0.05).

## Data Availability

The descriptive data used to support the findings of this study are restricted by the Ethical Committee of Dr. Soetomo General Academic Hospital Surabaya in order to protect patient privacy. Data are available from Cita Rosita Sigit Prakoeswa, cita-rosita@fk.unair.ac.id for researchers who meet the criteria for access to confidential data.

## References

[1] Bylund S., Kobyletzki L. B., Svalstedt M., Svensson Å (2020). Prevalence and Incidence of Atopic Dermatitis: A Systematic Review. *Acta Dermato-Venereologica*.

[2] Giam Y. C., Hebert A. A., Dizon M. V. (2016). A Review on the Role of Moisturizers for Atopic Dermatitis. *Asia Pacific Allergy*.

[3] Lisante T. A., Nuñez C., Zhang P. (2017). Efficacy and Safety of an Over-the-Counter 1% Colloidal Oatmeal Cream in the Management of Mild to Moderate Atopic Dermatitis in Children: A Double-Blind, Randomized, Active-Controlled Study. *Journal of Dermatological Treatment*.

[4] Coondoo A., Phiske M., Verma S., Lahiri K. (2014). Side-Effects of Topical Steroids: A Long Overdue Revisit. *Indian Dermatology Online Journal*.

[5] Umborowati M. A., Nurasrifah D., Indramaya D. M., Anggraeni S., Damayanti D., Prakoeswa C. R. S. (2020). The Role of Ceramide, Menthol and Polidocanol on Pruritus, Skin Barrier Function, and Disease Severity of Mild Atopic Dermatitis. *Journal of Pakistan Association of Dermatologists*.

[6] Simpson E. L., Leung D. Y. M., Eichenfield L. F., Boguniewicz M., Kang S., Amagai M., Bruckner A. L. (2019). Atopic Dermatitis. *Fitzpatrick’s Dermatology*.

[7] Raimondo A., Lembo S. (2021). Atopic Dermatitis: Epidemiology and Clinical Phenotypes. *Dermatology Practical and Conceptual*.

[8] Tanei R., Hasegawa Y. (2016). Atopic Dermatitis in Older Adults: A Viewpoint From Geriatric Dermatology. *Geriatrics and Gerontology International*.

[9] Pugliarello S., Cozzi A., Gisondi P., Girolomoni G. (2011). Phenotypes of Atopic Dermatitis. *Journal Der Deutschen Dermatologischen Gesellschaft*.

[10] Guglielmo A., Sechi A., Patrizi A., Gurioli C., Neri I. (2021). Head and Neck Dermatitis, a Subtype of Atopic Dermatitis Induced by Malassezia Spp: Clinical Aspects and Treatment Outcomes in Adolescent and Adult Patients. *Pediatric Dermatology*.

[11] Anggraeni S., Damayanti D., Umborowati M. A. (2022). Efficacy and Safety of Specific Immunotherapy With Aeroallergens in the Management of Atopic Dermatitis. *International Journal of Health Sciences*.

[12] Russo F., Milanesi N., Iannone M. (2020). Tuscan Consensus on the Diagnosis, Treatment and Follow up of Adult Atopic Dermatitis. *Giornale Italiano di Dermatologia e Venereologia*.

[13] Nurasrifah D., Umborowati M. A., Indramaya M., Zulkarnain I., Prakoeswa C. R. S. (2019). Efficacy of Ceramide, Menthol, and Polidocanol Compared to Petrolatum Jelly Toward Severity of Mild Atopic Dermatitis. *Berk Ilmu Kesehat Kulit Kelamin*.

[14] Wollenberg A., Kinberger M., Arents B. (2022). European Guideline (EuroGuiDerm) on Atopic Eczema-Part II: Non-Systemic Treatments and Treatment Recommendations for Special AE Patient Populations. *Journal of the European Academy of Dermatology and Venereology*.

[15] Danby S. G., Andrew P. V., Taylor R. N. (2022). Different Types of Emollient Cream Exhibit Diverse Physiological Effects on the Skin Barrier in Adults With Atopic Dermatitis. *Clinical and Experimental Dermatology*.

[16] Bradshaw L. E., Wyatt L. A., Brown S. J. (2023). Emollients for Prevention of Atopic Dermatitis: 5-Year Findings From the BEEP Randomized Trial. *Allergy*.

[17] Varothai S., Nitayavardhana S., Kulthanan K. (2013). Moisturizers for Patients With Atopic Dermatitis. *Asian Pacific Journal of Allergy & Immunology*.

[18] Angelova-Fischer I., Neufang G., Jung K., Fischer T. W., Zillikens D. (2014). A Randomized, Investigator-Blinded Efficacy Assessment Study of Stand-Alone Emollient Use in Mild to Moderately Severe Atopic Dermatitis Flares. *Journal of the European Academy of Dermatology and Venereology*.

[19] Sánchez-Quesada C., López-Biedma A., Toledo E., Gaforio J. J. (2018). Squalene Stimulates a Key Innate Immune Cell to Foster Wound Healing and Tissue Repair. *Evidence-based Complementary and Alternative Medicine*.

[20] Lin T. K., Zhong L., Santiago J. L. (2017). Anti-Inflammatory and Skin Barrier Repair Effects of Topical Application of Some Plant Oils. *International Journal of Molecular Sciences*.

[21] Marinho A., Nunes C., Reis S. (2021). Hyaluronic Acid: A Key Ingredient in the Therapy of Inflammation. *Biomolecules*.

[22] Damayanti U. M. A., Anggraeni S., Prakoeswa C. R. S., Cita Rosita Sigit Prakoeswa (2021). The Role of Aloe Vera and *Centella asiatica* to the Improvement of Skin Barrier Function in Indonesian Batik Workers. *Indian Journal of Forensic Medicine & Toxicology*.

[23] Diana I. A., Boediardja S. A., Sugito T. L. (2014). *Panduan Diagnosis Dan Tatalaksana Dermatitis Atopik Di Indonesia*.

[24] Yoshida T., Beck L. A., de Benedetto A. (2022). Skin Barrier Defects in Atopic Dermatitis: From Old Idea to New Opportunity. *Allergology International*.

[25] Otokpa G., Ibekwe P., Ukonu B., Ogunbiyi A. (2019). Skin Hydration in Adult Atopic Dermatitis Using Corneometry. *24th World Congress of Dermatology Abstract Book*.

[26] Zainal H., Jamil A., Md Nor N., Tang M. M. (2020). Skin pH Mapping and Its Relationship With Transepidermal Water Loss, Hydration and Disease Severity in Adult Patients With Atopic Dermatitis. *Skin Research and Technology*.

[27] Wakeman M. P. (2017). An Open-Label Forearm-Controlled Pilot Study to Assess the Effect of a Proprietary Emollient Formulation on Objective Parameters of Skin Function of Eczema-Prone Individuals Over 14 Days. *Clinical, Cosmetic and Investigational Dermatology*.

[28] Umborowati M. A., Anggraeni S., Damayanti P. C. R. S. (2022). The Beneficial Effect of Aloe Vera in Skin Barrier Function Improvement: A Double-Blind Randomized Trial of Madurese Batik Craftswomen. *Journal of Pakistan Association of Dermatologists*.

[29] Anggraeni S., Umborowati M. A., Damayanti D., Endaryanto A., Prakoeswa C. R. S. (2021). Role of *Centella Asiatica* and Ceramide in Skin Barrier Improvement: A Double Blind Clinical Trial of Indonesian Batik Workers. *Journal of Basic and Clinical Physiology and Pharmacology*.

[30] Akihisa T., Kojima N., Kikuchi T. (2010). Anti-Inflammatory and Chemopreventive Effects of Triterpene Cinnamates and Acetates From Shea Fat. *Journal of Oleo Science*.

[31] Purnamawati S., Indrastuti N., Danarti R., Saefudin T. (2017). The Role of Moisturizers in Addressing Various Kinds of Dermatitis: A Review. *Clinical Medicine and Research*.

[32] Bae M. J., See H. J., Choi G., Kang C. Y., Shon D. H., Shin H. S. (2016). Regulatory T Cell Induced by Poria Cocos Bark Exert Therapeutic Effects in Murine Models of Atopic Dermatitis and Food Allergy. *Mediators of Inflammation*.

[33] Chao C. L., Wang C. J., Huang H. W. (2021). *Poria Cocos* Modulates Th1/Th2 Response and Attenuates Airway Inflammation in an Ovalbumin-Sensitized Mouse Allergic Asthma Model. *The Life*.

[34] Choi E., Kang Y. G., Hwang S. H. (2019). In Vitro Effects of Dehydrotrametenolic Acid on Skin Barrier Function. *Molecules*.

[35] Paller A., Nardi R., Do H., Reha A., Viereck C., Castelli J. (2017). An Investigation Into Multifaceted Mechanisms of Action of Allantoin in Wound Healing. *Journal of the American Academy of Dermatology*.

[36] Vanessa V. V., Wan Ahmad Kammal W. S. L., Lai Z. W., How K. N. (2022). A Review of Moisturizing Additives for Atopic Dermatitis. *Cosmetics*.

